# Prostate‐specific antigen modulates the osteogenic differentiation of MSCs via the cadherin 11‐Akt axis

**DOI:** 10.1002/ctm2.27

**Published:** 2020-05-15

**Authors:** Longxiang Wu, Shiqi Xiang, Xiheng Hu, Miao Mo, Cheng Zhao, Yi Cai, Shiyu Tong, Huichuan Jiang, Linxiao Chen, Zhi Wang, Wei Xiong, Zhenyu Ou

**Affiliations:** ^1^ Department of Urology Xiangya Hospital of Central South University Changsha P.R. China; ^2^ Department of Orthopedics The Second Xiangya Hospital of Central South University Changsha P.R. China

**Keywords:** prostate‐specific antigen, mesenchymal stem cell, osteogenesis, prostate cancer

## Abstract

**Background:**

A high prevalence of osteoblastic bone metastases is characteristic of prostate cancer. Prostate‐specific antigen (PSA) is a serine protease uniquely produced by prostate cancer cells and is an important serological marker for prostate cancer. However, whether PSA modulates the osteogenic process remains largely unknown. In this study, we explored the effect of PSA on modulating the osteoblastic differentiation of mesenchymal stem cells (MSCs). In this study, we used flow cytometry, CCK‐8 assay, Alizarin red S (ARS) staining and quantification, alkaline phosphatase (ALP) activity and staining, Western blotting, and quantitative real‐time PCR (qRT‐PCR) to explore the effect of PSA on osteogenic differentiation of MSCs.

**Results:**

We first demonstrated that although PSA did not affect the proliferation, morphology, or phenotype of MSCs, it significantly promoted the osteogenic differentiation of MSCs in a concentration‐dependent manner. Furthermore, we demonstrated that PSA promoted the osteogenic differentiation of MSCs by elevating the expression of Cadherin 11 in MSCs and, thus, activating the Akt signaling pathway.

**Conclusions:**

In conclusion, we demonstrated that PSA could promote the osteogenesis of MSCs through Akt signaling pathway activation by elevating the expression of cadherin‐11 in MSCs. These findings imply a possible role of PSA in osteoblastic bone metastases in prostate cancer.

## 1 BACKGROUND

Patients with advanced prostate cancer (PCa) frequently have poor prognoses because of metastasis.^1^, ^2^, ^3^ The skeleton is one of the most common locations for prostate cancer metastasis.^2^, ^4^, ^5^ Bone metastases of prostate cancer are frequently characterized as osteoblastic (bone‐forming), resulting in substantial complications—including bone pain, fractures, spinal cord compression, and even hemiparesis—and leading to morbidity with no curative treatment.^4^, ^5^, ^6^ Although several studies have explored the possible mechanism of the above phenomenon, the precise pathogenesis of osteoblastic bone metastases in prostate cancer remains largely unknown.

Prostate‐specific antigen (PSA) is a widely used serological marker for prostate cancer.^7^, ^8^, ^9^ However, the physiological functions of PSA are largely unknown. PSA is a serine protease that can cleave parathyroid hormone‐related peptide, resulting in abolition of the ability of parathyroid hormone‐related peptide to stimulate cyclic AMP production, which in turn leads to a decrease in bone resorption.^10^, ^11^ Moreover, several studies have demonstrated that PSA can induce osteoblastic gene expression in human osteosarcoma cells, indicating that PSA might stimulate bone formation instead of decreasing bone resorption.^12^ However, the precise role of PSA in osteoblastic bone metastases in prostate cancer remains unclear.

Bone marrow‐derived mesenchymal stem cells (BM‐MSCs) are the main source of osteoblasts in vivo.^13^, ^14^, ^15^, ^16^, ^17^ Previous studies have demonstrated that bone‐metastatic prostate carcinoma favors MSC differentiation toward osteoblasts, a possible mechanism underlying osteoblastic bone metastasis in prostate cancer.^18^ However, whether PSA plays a role during this process has never been explored.

Taken together, our findings in this study demonstrate that PSA can promote the osteogenesis of MSCs through activation of the Akt signaling pathway by elevating the expression of cadherin‐11 in MSCs. This data may provide further insight into the pathogenesis of osteoblastic bone metastases in prostate cancer.

## 2 MATERIALS AND METHODS

### 2.1 MSC isolation and culture

This study was approved by the ethics committee of Xiangya Hospital of Central South University, Changsha, P.R. China. Nine healthy donors were recruited into the study. After being informed of the possible risks, all donors provided written informed consent. Bone marrow aspiration was performed, and the bone marrow samples were immediately processed. MSCs from the bone marrow samples were isolated using a density gradient centrifugation method. Briefly, the bone marrow samples were diluted in low‐glucose DMEM (GIBCO) containing 10% fetal bovine serum (FBS) (GIBCO). Mononuclear cells were prepared by gradient centrifugation at 900 × *g* for 30 min on a Percoll (Pharmacia Biotech) gradient with a density of 1.073 g/mL. The cells were washed and seeded (2 × 10^6^ cells/cm^2^) in 25 cm^2^ flasks containing low‐glucose DMEM supplemented with 10% FBS and cultured at 37°C in 5% CO_2_. The medium was replaced, and the cells in suspension were removed at 48 h every 3 or 4 days thereafter. MSCs were passaged when the culture reached 90% confluency. Cells were used for experiments at passage 3.

### 2.2 Flow cytometry

MSCs were digested and washed with phosphate‐buffered saline (PBS). After resuspension in PBS, MSCs were incubated with antibodies against CD29, CD44, CD105, CD14, CD45, and HLA‐DR (BD Pharmingen). MSC phenotypes were assessed using a BD Influx cell sorter (BD Biosciences) for cell identification.

### 2.3 Cell Counting Kit‐8 (CCK‐8) assay

MSCs in DMEM containing 10% FBS were seeded in 96‐well plates. PSA (R&D) was added at concentrations of 100, 250, and 500 ng/mL. After culture for 1, 3, 5, 7, and 9 days, the medium was removed, and the cells were incubated in 100 µL of fresh serum‐free DMEM containing 10 µL of CCK‐8 solution (Dojindo) at 37°C for 2 h. The absorbance was measured at 450 nm in a Varioskan Flash Spectral Scanning Multimode Reader (Thermo Fisher Scientific Inc).

### 2.4 Osteogenic differentiation assay

Osteogenic differentiation medium was prepared from DMEM supplemented with 10% FBS, 100 IU/mL penicillin, 100 IU/mL streptomycin, 0.1 µM dexamethasone (Sigma), 10 mM β‐glycerol phosphate (Sigma), and 50 µM ascorbic acid (Sigma). MSCs were seeded in 12‐well plates and cultured in osteogenic differentiation medium. The medium was replaced every 3 days. In some experiments, PSA was added at concentrations of 100, 250, and 500 ng/mL.

### 2.5 Stimulation of MSCs with prostate‐specific antigen (PSA)

The PSA were purchased from R&D Systems (1344‐SE‐010). According to previous studies, Prostate cancer patients with bone metastases are characteristic of high level of PSA (>100 ng/mL) in serum.^19^ Thus, we stimulate MSCs at a final concentration from 100 to 500 ng/mL.

### 2.6 Alizarin red S (ARS) staining and quantification

MSCs undergoing osteogenic differentiation were fixed in 4% paraformaldehyde and stained with 1% ARS (pH 4.3, Sigma) for 15 min. The stained MSCs were visualized after three washes with PBS. MSCs were destained with 10% cetylpyridinium chloride monohydrate (CPC, Sigma) for 1 h. A 200 µL aliquot was transferred to a 96‐well plate, and the absorbance was measured at 562 nm.

### 2.7 Alkaline phosphatase (ALP) activity and staining

For ALP staining, MSCs undergoing osteogenic differentiation were fixed, stained, and visualized as described above. The ALP staining assay was performed using an ALP kit (Sigma) according to the protocol.

ALP activity was detected using an ALP activity kit (Nanjing Jiancheng Biotech).

MSCs were lysed in RIPA lysis buffer (Thermo Fisher). The lysate was centrifuged at 12 000 rpm and 4°C for 30 min. Then, 100 µL of supernatant was incubated with 50 µL of reaction buffer at 37°C for 15 min. Color development was stopped with 150 µL of stop solution, and the absorbance was measured at 405 nm. The protein concentration in the lysate was determined using a BCA protein assay kit (Thermo Fisher) according to the protocol. ALP activity was ultimately expressed as units per gram of protein per 15 min (U/gpro/15 min).

### 2.8 Western blotting

Protein was extracted from MSCs and quantified as described above. Equal amounts of protein were separated by sodium dodecyl sulfate‐polyacrylamide gel electrophoresis and transferred to polyvinylidene difluoride (PVDF) membranes (Millipore). The polyvinylidene difluoride (PVDF) membranes were incubated with primary antibodies against GAPDH, OCN, Runx2, AKT, p‐AKT, Smad1, p‐Smad1/5/9, total catenin, p‐catenin, ERK1/2, and p‐ERK1/2 (all diluted 1:1000, Cell Signaling Technology) for 24 h and were then washed and incubated with a horseradish peroxidase (HRP)‐conjugated secondary antibody (1:3000, Cell Signaling Technology) for 1 h. Specific antibody‐antigen complexes were detected using Immobilon Western Chemiluminescent horseradish peroxidase (HRP) Substrate (Millipore).

### 2.9 AKT signaling pathway blockade assay

To inhibit AKT signaling pathway activation, AZD5363 (Selleck) was added to the medium at a concentration of 5 µM. The related experiments were performed on day 10 of induction.

### 2.10 Quantitative real‐time PCR (qRT‐PCR)

Total RNA was extracted using TRIzol reagent (Life Technologies), and cDNA was synthesized using PrimeScript^TM^ RT reagent kits (TaKaRa). qRT‐PCR was performed in a LightCycler^®^ 480 PCR system (Roche) using SYBR^®^ Premix Ex Taq^TM^ kits (TaKaRa) according to the protocol. The relative expression levels of each gene were analyzed using the 2^−ΔΔ^ *^Ct^* method and normalized to GAPDH expression. The sequences of the forward and reverse primers for each gene are shown below (Table 1).

### 2.11 Lentivirus production and infection

Lentiviruses encoding short hairpin RNA (shRNA) targeting Cadherin 11 (CDH11) (Lv) were constructed with a target sequence of 5`‐GGAGAACTACTGTTTACAAGC‐3′. The sequence of the negative control (NC) short hairpin RNA (shRNA) was 5′‐TTCTCCGAACGTGTCACGTTTC‐3′. In brief, lentivirus was produced by cotransfecting pGLVH1/GFP/Puro and packaging plasmids (pGag/Pol, pRev and pVSV‐G) into 293T cells. The culture supernatants containing Lvs were filtered and concentrated 72 h after transfection. Lvs (10^9^ TU/mL) and polybrene (5 µg/mL) were added to the medium and incubated with MSCs for 24 h at a multiplicity of infection (MOI) of 50. Related experiments were performed on day 10 of incubation.

### 2.12 Statistical analysis

All data are expressed as the means ± standard deviations (*SD*s). *t*‐tests and one‐way analysis of variance followed by the Bonferroni test and Pearson correlation test were performed for statistical analyses in SPSS (SPSS Inc). *P*‐values of less than .05 were considered statistically significant.

## 3 RESULTS

### 3.1 PSA did not affect the proliferation, morphology, or phenotype of MSCs

**FIGURE 1:**
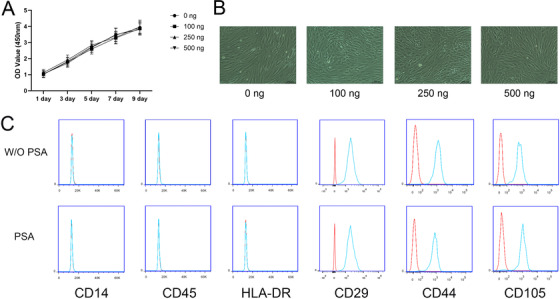
PSA did not affect the proliferation, morphology or phenotype of MSCs. (A) The OD values from CCK‐8 assays were equal in MSCs treated with 100‐500 ng/mL PSA. (B) The morphology of MSCs treated with 100‐500 ng/mL PSA was similar. (C) MSCs treated or not treated with 250 ng/mL PSA were positive for CD29, CD44, and CD105 and negative for CD14, CD45, and HLA‐DR (n = 3 independent experiments with three different MSC lines)

To evaluate the effect of PSA on the characteristics of MSCs, we stimulated MSCs using PSA at different concentrations to observe the changes in their proliferation ability, morphology, and phenotype. CCK‐8 assays showed that 100‐500 ng/mL PSA did not alter the proliferation of MSCs (Figure 1A). In addition, the morphology of MSCs was similar under stimulation with 100‐500 ng/mL PSA (Figure 1B). Moreover, whether cultured with or without PSA (250 ng/mL), MSCs were positive for CD29, CD44, and CD105 and negative for CD14, CD45, and HLA‐DR—showing the typical MSC phenotype (Figure 1C).

### 3.2 PSA accelerated the osteogenic differentiation of MSCs

**FIGURE 2:**
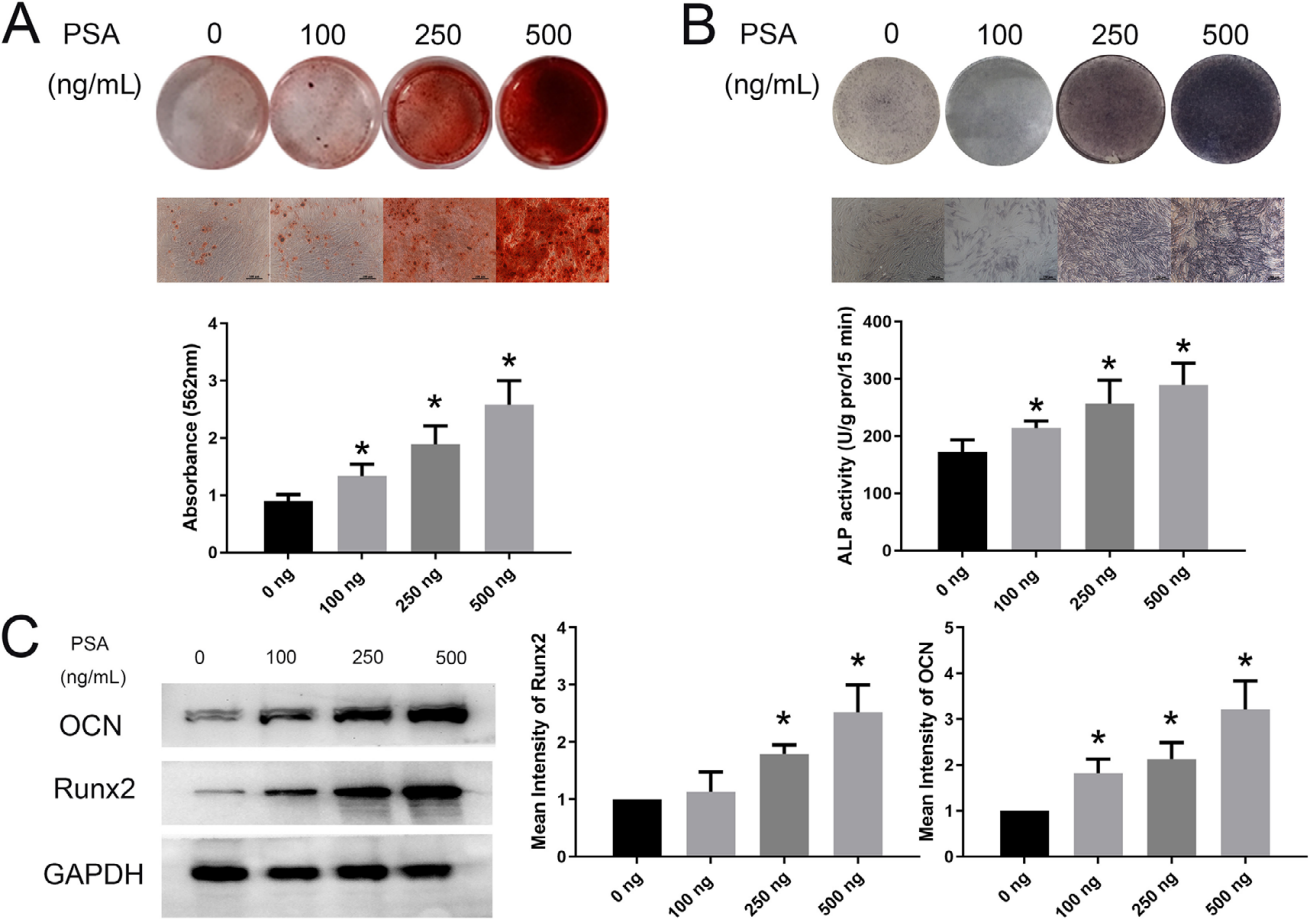
PSA accelerated the osteogenic differentiation of MSCs. (A) With increasing concentrations of PSA, the ARS staining intensity on day 14 of osteogenic induction increased. The quantification of ARS staining gradually increased from 0 to 500 ng/mL PSA. (B) The ALP staining intensity also increased after treatment with increasing concentrations of PSA. ALP activity was increased after PSA treatment. (C) Runx2 expression in MSCs was increased after stimulation with 250 and 500 ng/mL PSA, and OCN expression was increased after treatment with 100‐500 ng/mL PSA. * indicates *P* < .05 (n = 3 independent experiments with three different MSC lines)

Then, we sought to determine whether PSA affected the osteogenic differentiation ability. As shown by the results of ARS staining and quantification assays, PSA significantly promoted the osteogenic differentiation ability of MSCs on day 14 of osteogenic induction as the concentration increased from 100 to 500 ng/mL (Figure 2A). Consistent results were also obtained in the ALP assays (Figure 2B). Runx2 is an early marker of osteogenesis, and OCN is a marker of the later period. As the stimulatory concentration of PSA increased, the protein expression of OCN increased markedly. The expression of Runx2 followed a similar trend, except for 100 ng/mL PSA (Figure 2C).

### 3.3 PSA activated the AKT signaling pathway in MSCs during osteogenic differentiation

**FIGURE 3:**
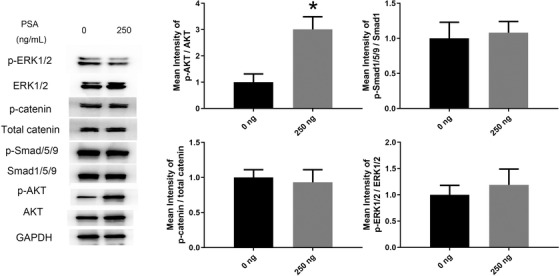
PSA activated the AKT signaling pathway in MSCs during osteogenic differentiation. The phosphorylation levels of molecules in the AKT signaling pathway were significantly increased under stimulation with 250 ng/mL PSA. The phosphorylation levels of molecules in the ERK, Smad1/5/9, and catenin signaling pathways were unchanged after PSA treatment. * indicates *P *< .05 (n = 3 independent experiments with three different MSC lines)

Previous studies have demonstrated that the osteogenic differentiation ability of MSCs is regulated by numerous signaling pathways including the BMP/Smad, WNT, MAPK, and AKT signaling pathways. Western blot assays on day 10 of induction showed that the level of the AKT signaling pathway activation in MSCs was significantly increased after stimulation with 250 ng/mL PSA (Figure 3). Surprisingly, the levels of ERK, Smad1/5/9, and Catenin signaling pathway activation were unchanged with PSA stimulation (Figure 3). These results showed that PSA affected MSC osteogenesis through the AKT signaling pathway.

### 3.4 AZD5363 reversed the facilitating effect of PSA on MSC osteogenesis

**FIGURE 4:**
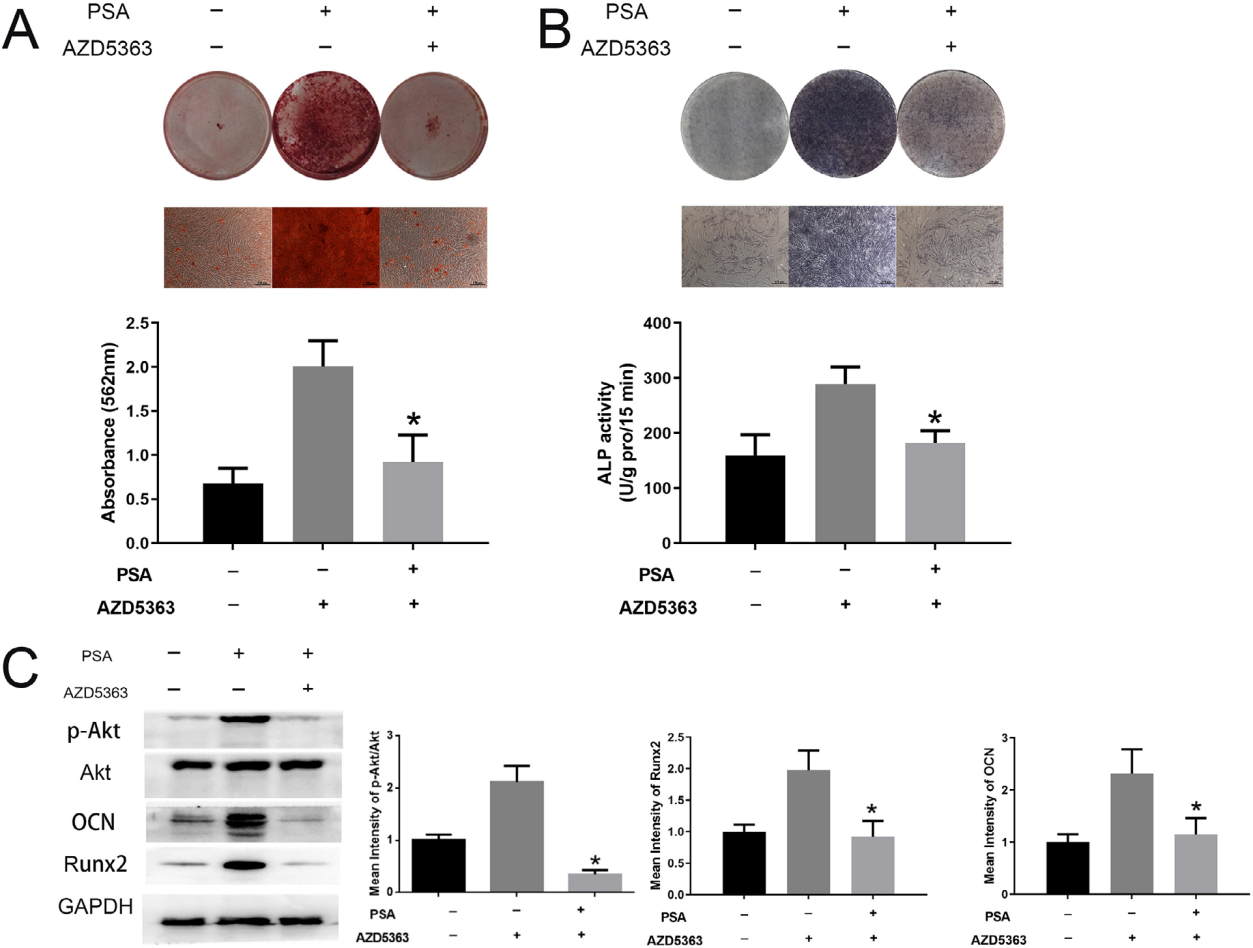
AZD5363 reversed the facilitating effect of PSA on MSC osteogenesis. (A) After treatment with AZD5363, ARS staining and quantification were reduced to normal levels compared to those in the group treated only with PSA. (B) AZD5363 also weakened the ALP staining intensity and reduced ALP activity in MSCs treated with PSA on day 14 of induction. (C) AZD5363 treatment significantly inhibited AKT signaling pathway activation. In addition, the expressions of both Runx2 and OCN were reduced by AZD5363. * indicates *P *< .05 (n = 3 independent experiments with three different MSC lines)

To further confirm the role of the AKT signaling pathway in the osteogenesis‐promoting effect of PSA, AZD5363, an inhibitor of the AKT signaling pathway, was added during osteogenic induction.^20^ The osteogenic differentiation ability of MSCs treated with PSA was noticeably inhibited by AZD5363, as shown by the ARS assay (Figure 4A) and ALP assay (Figure 4B). In addition, the levels of the osteogenesis markers Runx2 and OCN were decreased to normal levels by PSA (Figure 4C). In addition, the phosphorylation (activation) levels of molecules in the AKT signaling pathway were also decreased by AZD5363, as previously reported (Figure 4C). These results confirmed the critical role of the AKT signaling pathway in the facilitating effect of PSA on MSC osteogenesis.

### 3.5 PSA promoted the expression of Cadherin 11 in MSCs during osteogenic differentiation

**FIGURE 5:**
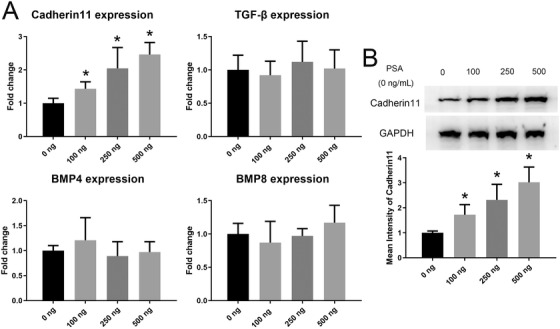
PSA promoted the expression of Cadherin 11 in MSCs during osteogenic differentiation. (A) CDH11 gene expression gradually increased after treatment with 100‐500 ng/mL PSA. The expression levels of BMP4, BMP8, and TGFβ were unchanged after PSA treatment. (B) CDH11 expression at the protein level was also enhanced after PSA treatment on day 10 of induction. * indicates *P* < .05 (n = 3 independent experiments with three different MSC lines)

A previous study demonstrated that PSA can significantly induce several osteogenesis‐induced genes, including CDH 11, BMP4, BMP8 and TGFβ, in osteosarcoma SaOS‐2 cells.^12^ Thus, we assumed that PSA may induce the osteogenesis of MSCs through one or several of the aforementioned factors. As shown by qRT‐PCR, the expression of CDH11 mRNA in MSCs on day 10 of osteogenic induction was dramatically elevated with PSA stimulation. The increase in CDH11 expression followed the gradually increasing concentration of PSA (Figure 5A). However, the expression levels of BMP4, BMP8, and TGFβ were unchanged with PSA stimulation (Figure 5A). Moreover, PSA promoted the protein expression of CDH11 in MSCs during osteogenic differentiation (Figure 5B).

### 3.6 Lv‐CDH11 reversed the PSA‐induced AKT activation and osteogenic program in MSCs

**FIGURE 6:**
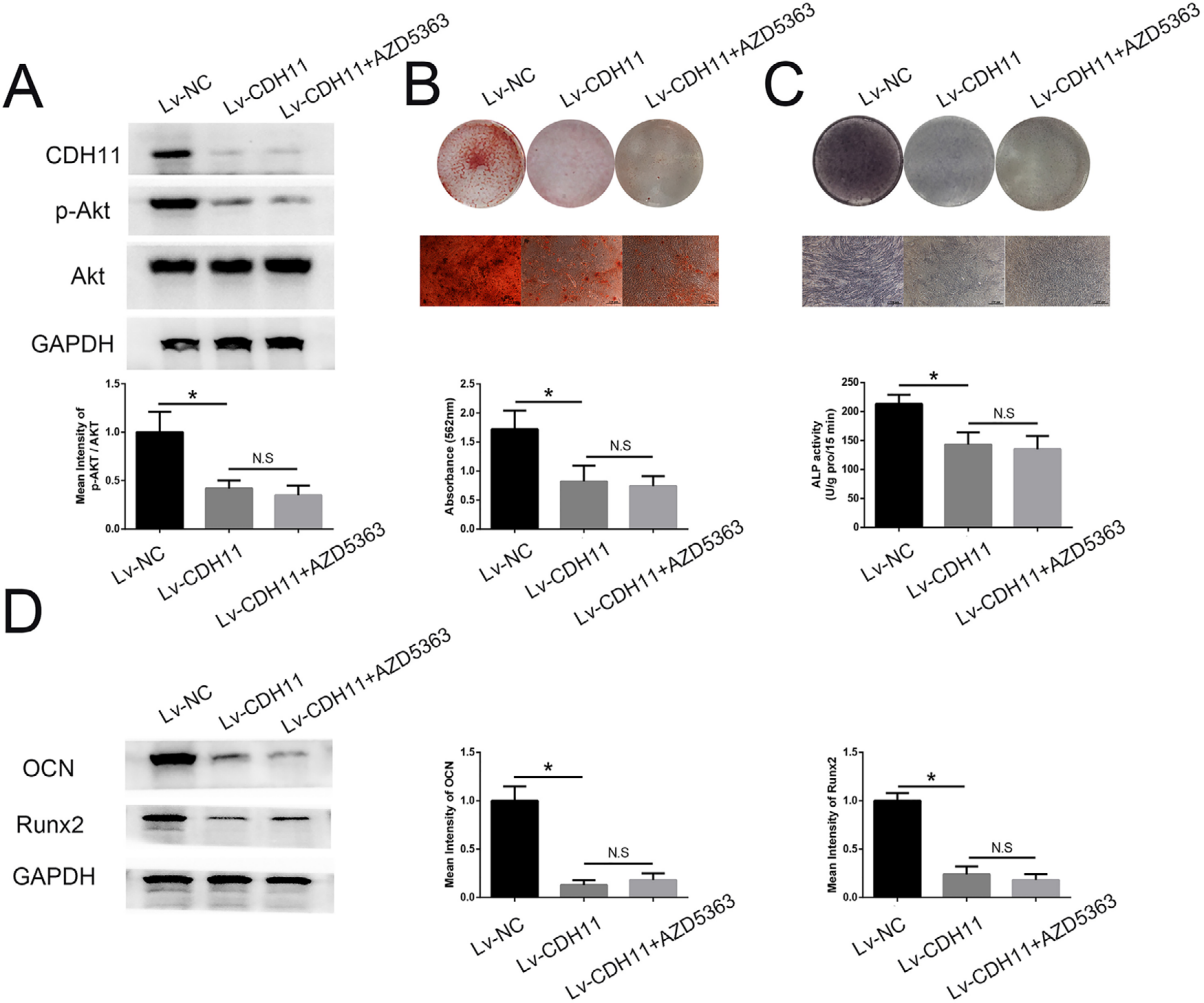
Lv‐CDH11 reversed the PSA‐induced AKT activation and osteogenic program in MSCs. (A) The phosphorylation levels of molecules in the AKT signaling pathway were decreased by Lv‐CDH11 compared to control lentivirus on day 10 of induction under PSA treatment, adding Lv‐CDH11 with AZD5363 together could not further inhibit the AKT activation. (B) Lv‐CDH11 reversed ARS staining and quantification in MSCs treated with PSA, adding Lv‐CDH11 with AZD5363 together could not further inhibit the ARS staining and quantification. (C) Lv‐CDH11 reversed ALP staining and activity in MSCs treated with PSA, adding Lv‐CDH11 with AZD5363 together could not further inhibit the ALP staining and activity. (D) Runx2 and OCN expression in MSCs treated with PSA was reduced by Lv‐CDH11, adding Lv‐CDH11 with AZD5363 together could not further inhibit the Runx2 and OCN expression. * indicates *P* < .05 (n = 3 independent experiments with three different MSC lines)

Lv‐CDH11 was generated to confirm the role of CDH11 in PSA‐induced AKT activation and subsequent osteogenesis in MSCs. Remarkably, the level of the AKT signaling pathway activation in MSCs transfected with Lv‐CDH11 was lower than that in MSCs transfected with control lentivirus under stimulation with 250 ng/mL PSA and adding Lv‐CDH11 with AZD5363 together could not further inhibit the AKT activation (Figure 6A). Moreover, although both groups were cultured with PSA, the osteogenic differentiation ability of MSCs in the Lv‐CDH11 group was reduced compared to that in the control lentivirus group and adding Lv‐CDH11 with AZD5363 together could not further inhibit the osteogenic differentiation capacity of MSCs (Figures 6B and 6C). The Runx2 and OCN expression levels were consistent with the results of the ARS and ALP assays (Figure 6D). Besides, adding AZD5363 showed no effect on the expression of CDH11 of MSCs (Supporting Information Figure 1). Collectively, these results demonstrated that PSA promoted CDH11 expression in MSCs, which in turn activated the AKT signaling pathway and subsequently accelerated MSC osteogenesis.

## 4 DISCUSSION

In this study, we showed that although PSA did not affect the proliferation, morphology, or phenotype of MSCs, it promoted the osteogenesis of MSCs. We further demonstrated that PSA elevated the expression of CDH11 11 at both the mRNA and protein levels and, in turn, activated the Akt signaling pathway in MSCs. These results indicate that PSA may promote the osteogenic differentiation of MSCs in bone and thus mediates osteoblastic bone metastases in prostate cancer.

A previous study demonstrated that approximately 26.5% of men have been diagnosed with prostate cancer worldwide, approximately 13.7% of whom were diagnosed within the Asia‐Pacific region.^2^, ^21^ The skeleton is the most common site of prostate cancer metastasis, as bone metastasis occurs in approximately 70% of men with advanced prostate cancer.^4^, ^22^ Bone metastases of prostate cancer are frequently characterized as osteoblastic, resulting in substantial complications—including bone pain, fractures, spinal cord compression, and even hemiparesis—and leading to morbidity with no curative treatment.^4^, ^23^ Moreover, prostate cancer‐induced bone metastases can, in turn, enhance prostate cancer cell growth and confer therapeutic resistance in the setting of bone metastasis.^4^, ^24^ The vicious cycle between prostate cancer and prostate cancer‐induced osteogenic lesions will seriously reduce not only the life span but also the quality of life of patients. Thus, determining the precise pathogenic mechanism underlying osteoblastic bone metastases in prostate cancer is highly important.

PSA is one of the most well‐known biomarkers for prostate cancer, but its precise role in bone metastasis is unclear.^8^, ^25^ Previous studies have confirmed that PSA levels are positively correlated with bone metastasis.^26^ However, we sought to determine whether PSA affects the induction of osteoblastic bone metastases, in addition to its role as a biomarker for bone metastasis, in prostate cancer. PSA is a serine protease that can cleave parathyroid hormone‐related peptide, resulting in abolition of the ability of parathyroid hormone‐related peptide to stimulate cyclic AMP production, which in turn leads to a decrease in bone resorption. The balance of bone remodeling is controlled by osteoblast activity and osteoclast activity.^27^ Whether abnormally increased osteoblast activity or decreased osteoclast activity leads to pathological osteogenesis is unknown. However, several studies have demonstrated that the mechanism of osteoblastic bone metastases in prostate cancer operates via increased osteoblast activity rather than decreased osteoclast activity.^28^, ^29^ Thus, we assume that although PSA can decrease osteoclast activity to inhibit bone resorption, it may also modulate osteogenic activity to promote osteoblastic bone metastases in prostate cancer. Indeed, Nadiminty et al confirmed that PSA can stimulate the expression of several osteogenesis‐related genes in osteosarcoma cells, indicating that PSA has a potential role in promoting the osteogenic process in vivo.^12^ MSCs were the major origin of osteoblast in vivo.^30^ Many studies have determined that enhanced ability of MSCs in osteogenic differentiation led to excessive bone formation in vivo.^31^, ^32^ So, we studied whether PSA could affect the osteogenic differentiation of MSCs. Through research, we confirmed that PSA accelerated MSCs osteogenesis via CDH11‐AKT axis. Based on these results, we suggest that metastasizing prostate cancer cells were located in bone and then secreted large amount of PSA, which promoted the osteogenic differentiation of MSCs and form osteoblastic metastasis focus.

However, how PSA exhibited its function on MSCs and increased the CDH11 followed by enhanced osteogenic differentiation were still unclear. As a serine protease, PSA has been reported to exhibits its functions through cleaving other functional proteins.^11^ Besides, PSA can also bind to other protein to form complexes, which interact with specific receptors on cell surface to activate signal pathway downstream.^33^ In our study, we found that PSA promoted the CDH11 expression, which activated the AKT signal pathway and then accelerated the osteogenic differentiation of MSCs. We suggest that PSA may exhibit its function on MSCs through two possible mechanisms. On one hand, PSA may bind to its receptors on MSCs and then increases the CDH11 expression. On the contrary, PSA may cleave some osteogenesis‐related factors, affect the functions of these factors and then promote the osteogenic differentiation of MSCs. However, which possible mechanisms predominate in MSCs, and through which receptors or factors PSA exhibit its functions are still unclear. These limitation need to be addressed in our future research.

The relationship between the Akt signaling pathway and prostate cancer has been thoroughly studied over the past decades.^34^, ^35^ Activation of the Akt pathway is observed more frequently as prostate cancer progresses toward a resistant, metastatic disease.^36^, ^37^ Biomarkers in this pathway have shown that activation of this pathway is correlated with high‐grade disease and an increased risk of disease progression.^35^ Additionally, PTEN is a negative regulator of Akt pathway activity.^38^ PTEN deletions and mutations that result in the expression of an inactive protein lead to increased Akt pathway activity.^38^ Mutations in the PTEN tumor suppressor are common events in prostate cancer; studies have shown loss of heterozygosity at the PTEN locus in up to 60% of prostate cancer samples.^39^, ^40^ Mice with homozygous PTEN knockout die in utero, while mice with prostate‐specific deletion of PTEN develop invasive prostate cancer.^39^ Moreover, the Akt signaling pathway acts as one of the most classic modulators in promoting the osteogenic process.^41^ The Akt pathway induces the osteogenic differentiation of MSCs through the canonical GSK‐3β–β‐catenin loop.^41^, ^42^ In our study, we demonstrated that PSA can significantly increase Akt activation in MSCs and, in turn, promote the osteogenic differentiation of MSCs. Inhibiting Akt pathway activation can reverse PSA‐induced osteogenesis of MSCs. Thus, the results of our study indicate that specific targeting of the Akt signaling pathway for prostate cancer therapy is very promising. Indeed, multiple small molecule inhibitors of the Akt pathway have been investigated in both in vitro and in vivo models of prostate cancer.^43^, ^44^

Nadiminty et al demonstrated that PSA could increase the levels of multiple osteogenesis genes, including BMP 4, BMP 8, CDH 11, and TGF‐β, in osteosarcoma cell lines.^12^ Consistent with that finding, we demonstrated that PSA significantly induced the expression of Cadherin‐11 in MSCs at both the mRNA and protein levels. CDH 11 is a member of the cadherin family and can regulate the differentiation of mesenchymal cells into cells of the osteolineage by inducing ALP and FGF receptor 2 expression, resulting in bone and tissue formation.^45^ Moreover, despite inducing the osteogenic differentiation of MSCs, CDH 11 also plays an important role in prostate cancer.^46^, ^47^ Sue‐Hwa Lin et al demonstrated that cadherin‐11 plays a critical role in some of the earliest steps in the metastasis of prostate cancer.^46^ This result remind us that except for promoting the osteoblastic metastasis focus, CDH11 may enhance the metastasis of prostate cancer cells to bone, which form a vicious cycle. However, this vicious cycle needs further studies. Therefore, cadherin‐11 may be a very valuable target for prostate cancer treatment, and further study of cadherin‐11 in prostate cancer may be highly valuable.

## 5 CONCLUSIONS

In conclusion, in this study, we demonstrated that PSA can promote the osteogenesis of MSCs through Akt signaling pathway activation by elevating the expression of cadherin‐11 in MSCs. These findings imply a possible role of PSA in mediating osteoblastic bone metastases in prostate cancer. To further explore the precise role of PSA in osteoblastic bone metastases in prostate cancer, in vivo gain‐ and loss‐of‐function experiments are highly important and will be the focus of our future research.

## CONFLICT OF INTEREST

The authors declare that they have no conflict of interest.

## Supporting information

Supporting Figure S1
