# Supplementary figures and images for: Prostate‐specific antigen modulates the osteogenic differentiation of MSCs via the cadherin 11‐Akt axis
Source: Clin Transl Med. 2020 May 15;10(1):363–73. doi: 10.1002/ctm2.27 (PMC7240859; doi:10.1002/ctm2.27)

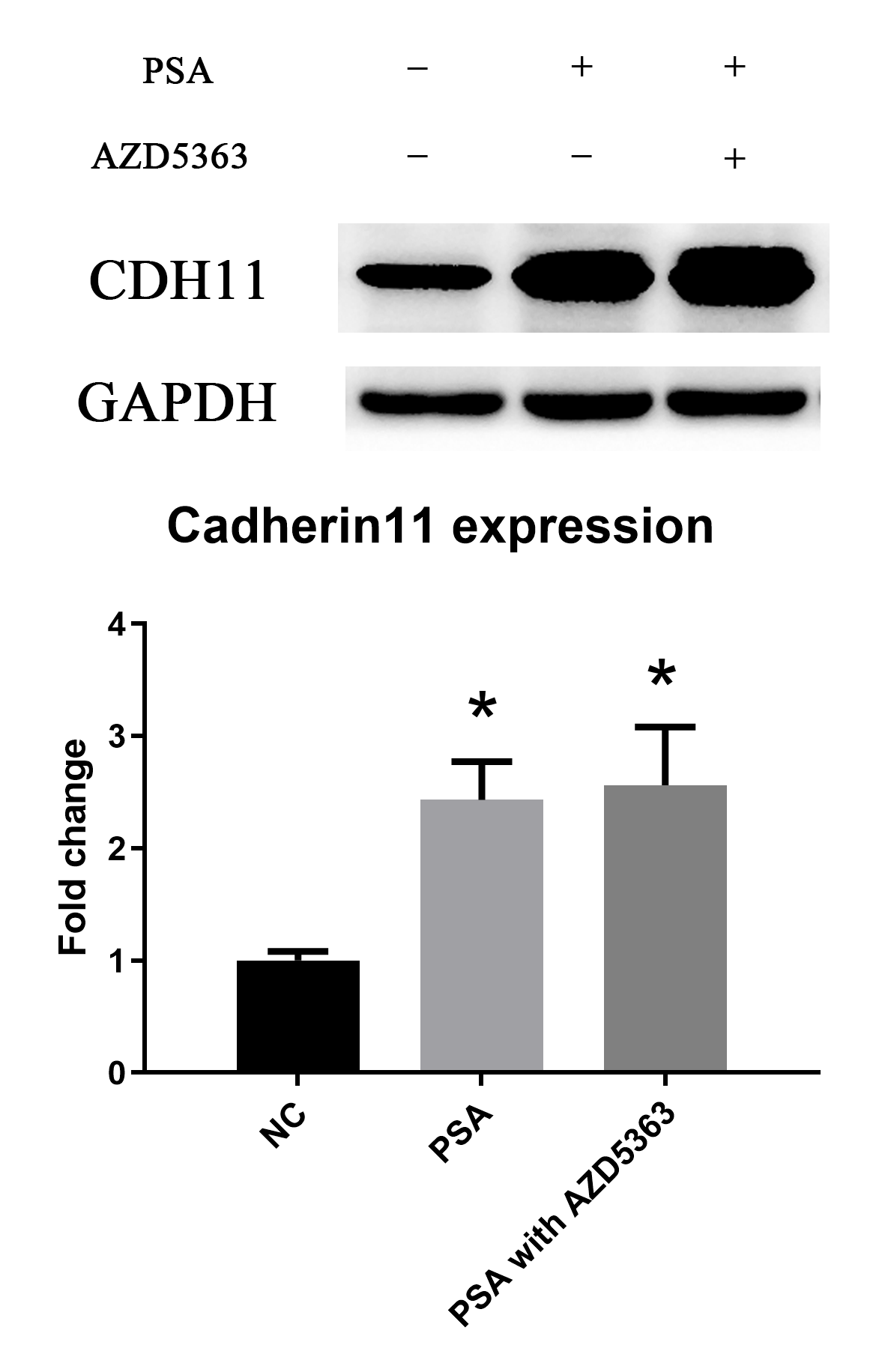

Supplement: Supplementary file 1 — Supporting Figure S1 [file CTM2-10-363-s001.tif]
